# The presence of antibiotic-resistant *Staphylococcus* spp. and *Escherichia coli* in smallholder pig farms in Uganda

**DOI:** 10.1186/s12917-020-02727-3

**Published:** 2021-01-18

**Authors:** K. Ikwap, E. Gertzell, I. Hansson, L. Dahlin, K. Selling, U. Magnusson, M. Dione, M. Jacobson

**Affiliations:** 1 Makerere University, College of Veterinary Medicine, Animal Resources and Biosecurity, Kampala, Uganda; 2 Swedish University of Agricultural Sciences, Faculty of Veterinary Medicine and Animal Science, Uppsala, Sweden; 3 International Livestock Research Institute, Dakar, Senegal

**Keywords:** Anti-microbial resistance, Bacteria, Broth micro-dilution, MRSA, Swine, Veterinary medicine

## Abstract

**Background:**

The development of antimicrobial resistance is of global concern, and is commonly monitored by the analysis of certain bacteria. The aim of the present study was to study the antibiotic susceptibility in isolates of *Staphylococcus* spp. and *Escherichia* (*E*.) *coli* obtained from healthy pigs originating from nineteen herds enrolled in a study on herd health management in Lira district, northern Uganda. Skin and nasal swabs were analyzed for the presence of *Staphylococcus* spp., and selectively cultivated to investigate the presence of methicillin-resistant *Staphylococcus* (*S*.) *aureus* (MRSA), and rectal swabs were analyzed for the presence of *E. coli*. Antibiotic susceptibility was tested by broth micro-dilution. Information on the antibiotic usage and treatment regimens during the previous year was gathered using structured interviews and longitudinal data.

**Results:**

In *Staphylococcus* spp., resistance to penicillin (10/19 isolates; 53%), fusidic acid (42%) and tetracycline (37%) were most commonly found. In *E. coli*, resistance to sulfamethoxazole (46/52 isolates; 88%), tetracycline (54%) and trimethoprim (17%) was most frequent. Methicillin-resistant *S. aureus* was found in one sample (1/50; 2%). Multi-drug resistant isolates of *Staphylococcus* spp. and *E. coli* were found in 54 and 47% of the herds, respectively. At the herd level, no associations could be made between antibiotic resistance and herd size or treatment regimens for either of the bacteria.

**Conclusion:**

In conclusion, resistance to important antibiotics frequently used in animals in Uganda was common, and the presence of MRSA was demonstrated, in Ugandan pig herds.

**Supplementary Information:**

The online version contains supplementary material available at 10.1186/s12917-020-02727-3.

## Introduction

Antimicrobial resistance (AMR) is a threat to public as well as to animal health [1, 2]. In some bacterial species, the AMR may be intrinsic, but might also develop following the extensive use of antibiotics [3, 4]. Thus, high levels of antibiotic resistance generally correlates with high antibiotic usage [5, 6], and antibiotics are still used in many countries for regular prophylactic treatments or as growth promoters in food-producing animals [7].

Different *Staphylococcus* spp. are a part of the commensal microbiota on e.g. the skin and nares in healthy humans and animals [8, 9], but may also cause disease ranging from abscesses and mastitis to septicemia [10, 11]. In response to antibiotic treatment, the bacteria may develop resistance [12], and methicillin-resistant *Staphylococcus* (*S.*) *aureus* (MRSA) have emerged as one of the most common antibiotic-resistant pathogens worldwide [13]. Further, in the last decades, livestock-associated MRSA have been found in healthy domestic animals, including pigs, posing a risk especially to humans with occupational contact with livestock [9, 14].

To monitor the development of antibiotic resistance, indicator bacteria such as *Escherichia* (*E.*) *coli* may be used [15, 16]. *Escherichia coli* are commensal, or potentially opportunistic pathogens that are common in the intestinal tract of animals and humans. Besides being used as indicator bacteria, resistance in *E. coli* may be important since resistance genes may be transferred to other, pathogenic, strains of *E. coli*, or to other bacteria.

In Uganda, information on antibiotic usage and AMR in livestock is scarce due to the lack of national surveillance programs [17]. Most large pig farms routinely use antibiotic prophylactic treatments [18] but the usage does not seem to be as common in smallholder farms [19], where most of the Ugandan pigs are found [20]. While scientific studies on MRSA are scarce in Uganda [21], a previous study on pig herd health found antibiotic resistance in clinical isolates of *S. sciuri* and *E. coli* [22] and thus, further investigations were warranted.

The aim of this study was to investigate the presence and antibiotic resistance of *Staphylococcus* spp., including MRSA, and *E. coli* isolated from pigs in smallholder farms in Lira district, northern Uganda, by bacterial cultivation of swab samples and determination of antibiotic susceptibility by broth micro-dilution.

## Materials and methods

### Study design

The study was conducted in the four sub-counties closest to Lira town, Lira district, in the northern region of Uganda. A list of 250 pig farmers were obtained from the District Veterinary Office. From this list, 20 herds, meeting the inclusion criteria of having at least one sow and keeping the pigs confined or tethered, were selected by simple randomization. One farmer had sold his pigs prior to the visit, and thus, 19 herds remained in the study. As categorized by Ouma et al. [23], herds with less or equal to three sows/year were termed small, while those with more than three sows/year were termed large.

### Collection of data on the antibiotic usage

Information on the antibiotic usage during September 2018–September 2019 was gathered by several methods applied in all herds. In September 2019, a structured interview was conducted (see Additional file 1). In a separate study, the farms had previously been visited once a month for a period of one year [22]. During these visits, information on treatments and antibiotic usage was gathered by semi-structured interviews, including the reasons for the treatments, the drugs and dosage used, and the number of treatments, and by observations on the drugs available on the farms. Further, farmers were asked to continuously record treatment information, as also investigated through the interviews. The farmers’ records were constructed in the local language, and the questions and answers in the interviews were continuously translated to and from the local language by either of two local animal health workers, and were recorded in English.

Since information on the number of pigs treated at each occasion was often missing, a treatment occasion (TO) was defined as one reported antibiotic treatment in a herd, regardless of whether one or many pigs were treated. The number of treatment occasions per pig was calculated as the number of TO in a herd during September 2018 to September 2019, divided by the average number of pigs of all age categories in that herd during the same period.

The results were analyzed at herd level using Fisher’s exact test (https://epitools.ausvet.com.au/twobytwotable) comparing resistance (Yes/No) to any of the antibiotics included in the panels, resistance to single selected antibiotic substances (tetracycline and penicillin/ampicillin) and the presence of multi-drug resistance, to the herd size and treatment regimens (see Additional file 2).

### Sampling

Pigs of approximately two months of age were targeted for sampling of *Staphylococcus* spp., as MRSA is reported to be more common in this age category [24, 25]. In the sampling of *E. coli*, pigs of approximately six months of age were targeted, in accordance with previously reported surveillance programs [15]. If the targeted age-categories were not available at the sampling occasions, pigs as close to the desired age as possible were instead selected, giving a median age in the sampled pigs of five and seven months for *Staphylococcus* spp. and *E. coli*, respectively. The number of pigs sampled in each herd depended on the herd size, and the sampling was performed as described in Table 1.

**Table 1:** Sample size per herd for analysis of *E. coli* and *Staphylococcus* spp. in Lira, Uganda

Herd no.	Pigs (*n*)	Analyzed samples (*n*)	
		*E. coli*	*Staphylococcus* spp.*
1	7	2	2 (2)
2	9	3	3 (3)
3	18	3	3 (3)
4	3	1	1 (1)
5	126	10	7 (21)
6	4	2	1 (1)
7	2	1	1 (1)
8	16	3	4 (4)
9	15	3	4 (6)
10	25	4	4 (8)
11	30	4	5 (8)
12	1	1	1 (1)
13	15	3	3 (6)
14	3	1	1 (1)
15	5	2	2 (2)
16	8	3	1 (3)
17	0	0	0
18	98	5	5 (10)
19	3	1	1 (1)
20	1	1	1 (1)
	**Total**	**53**	**50**

*Analyzed samples include individual samples and samples that were pooled two-three per pen or group to increase the detection rate of Staphylococcus spp. The figures given within brackets indicate the total number of pigs sampled

For the analysis of *Staphylococcus* spp., each pig was sampled using a single swab (Transystem™, Copan Diagnostics Inc., Murrieta, California, USA) to swab the nasal cavity, behind the ears and around the perineum, as multiple-site sampling has been shown to increase the detection rate of MRSA [26]. When possible, additional individual samples were taken from the targeted age category, and later pooled two-three together per pen or group at the laboratory, to enhance the possibility of detecting MRSA [27]. Individual rectal swabs were taken from each pig for the analysis of *E. coli*. All swabs were transported on ice and stored in Amies medium with charcoal at + 4 to+ 10 °C until being cultivated at the District Veterinary Office Laboratory in Lira. However, since the incubator in Lira broke down towards the end of the farm visits, and to be able to perform all cultivation steps in one laboratory, samples collected during the final days of field work were instead stored at + 4 to + 10 °C and analyzed at the Central Diagnostic Laboratory at Makerere University in Kampala.

### Laboratory analyses

#### Bacterial cultivation of Staphylococcus spp.

Individual swabs, or two-three pooled swabs, were submerged in 10 mL non-selective Mueller Hinton broth (MHB; National Veterinary Institute, SVA, Uppsala, Sweden) within 0–6 days (median 2), and incubated for 24 h at 37 °C. The incubated broth was immediately analyzed for MRSA by a two-step enrichment method [28], and the remaining broth was stored at + 4 to + 10 °C before being analyzed for the presence of other *Staphylococcus* spp.

In the analysis of MRSA, 1 mL of the incubated MHB was transferred to 9 mL selective Tryptic Soy broth supplemented with azotrenam 72 mg/L and cefoxitin 3.8 mg/L (SVA, Uppsala, Sweden), and incubated for 24 h at 37 °C. The samples were vortexed and 20 μL were streaked onto selective chromogenic Oxoid Brilliance MRSA 2 agar (Oxoid, Basingstoke, England), and incubated at 37 °C. The growth was assessed after 24 and 48 h. If present, up to five suspected, light blue, colonies were streaked onto 5% bovine blood agar plates (SVA, Uppsala, Sweden), and incubated at 37 °C for 24 h. Pure-cultured isolates having a morphology suspected to be MRSA were tested for catalase production and KOH reactivity (SVA, Uppsala, Sweden). Isolates with the correct morphology, with α- and/or β-hemolysis, and that were catalase-positive and KOH-negative, were preliminary identified as MRSA.

The presence of additional *Staphylococcus* spp. were analyzed within 5–14 days (median 5) at the laboratory in Kampala, by culturing 20 μL from the remaining MHB onto blood agar plates supplemented with 5% bovine blood, and incubated at 37 °C for 24 h. From each sample, up to five suspected, opaque, white or yellow, medium-sized, colonies with or without hemolysis were cultivated on blood agar plates as described above. In the case of bacterial swarming, the sample was re-cultured onto Cysteine Lactose Electrolyte Deficient (CLED) agar plates (Oxoid, Basingstoke, England) since CLED agar is electrolyte (salt) deficient, and prevent the swarming of bacteria. The identification of *Staphylococcus* spp. was based on the colony morphology and the production of catalase, but no reaction in KOH.

##### Further identification of Staphylococcus spp

All isolates identified as *Staphylococcus* spp. were later transported to Sweden in Amies transport medium with charcoal and analyzed by Matrix-Assisted Laser Desorption/Ionization Time of Flight Mass Spectrometry (MALDI-TOF MS; Microflex LT System, Bruker Daltonik GmbH, Bremen, Germany) for species identification. Isolates presumed to be MRSA and confirmed as *S. aureus* by MALDI-TOF MS were also prepared according to Capurro et al. [29] and analyzed for the presence of *mecA*, *mecC*, *nuc* and *pvl* genes by a real-time quadruplex PCR according to Pichon et al. [30]. Briefly, pure-cultured colony material was suspended in lysostaphin, incubated at 37 °C for 10 min, proteinase K and Tris-HCL were added and the mixture was incubated at 54 °C for an additional 10 min. After boiling for 5 min, the samples were cooled and centrifuged, and the supernatant was frozen at − 20 °C before PCR. The PCR assay used a 20-μL reaction volume with 0.5 mM primers targeting the sequences according to Pichon et al. [30]. The amplification included an initial denaturation of 95 °C for 5 min., followed by 40 cycles of denaturation at 94 °C for 15 s. and 40 s. of annealing at 58 °C. *Staphylococcus aures* subsp. *aureus* CCUG 60578 (*nuc* positive, *mecA* positive, *pvl* positive) and *Staphylococcus aures* subsp. *aureus* CCUG 63582 (*nuc* positive, *mecA* negative, *mecC* positive) were used as quality control.

#### Bacterial cultivation of E. coli

Individual swabs were streaked on CLED agar plates within 0–4 days (median 0), and incubated for 24 h at 37 °C. Up to five suspected, greyish-white, opaque, medium-sized, lactose-fermenting colonies were re-cultured onto 5% horse blood agar plates (SVA, Uppsala, Sweden), and incubated at 37 °C for 24 h. The isolates were analyzed for biochemical reactions with oxidase (Becton, Dickinson and Company, Franklin Lakes, NJ, USA), KOH and the spot indole test (SVA, Uppsala, Sweden). Isolates with correct morphology, without hemolysis or displaying α-hemolysis, and that were oxidase-negative, indole-positive and KOH-negative, were identified as *E. coli*. One colony per sample was randomly selected for further analyses.

#### Antimicrobial susceptibility testing

Antimicrobial susceptibility testing of the *E. coli* isolates was performed by broth micro-dilution at the Makerere University, Kampala, and at the Swedish University of Agriculture on isolates of MRSA and other *Staphylococcus* spp. following identification by MALDI-TOF MS and PCR. Commercial plates for *Staphylococcus* spp. (VetMIC™ STAF/STREP, SVA, Uppsala, Sweden), and for *E. coli* (Sensititre™ EU Surveillance Salmonella/E. coli EUVSEC Plate, Thermo Fisher Scientific, Waltham, Massachusetts, USA) were used in the analyses according to the manufacturer’s instructions. Briefly, the colony material from one pure-cultured isolate was incubated in 5 mL of cation-adjusted Mueller Hinton broth (CAMHB; SVA, Uppsala, Sweden) for 3 h at 37 °C. The sample was vortexed and 10 μL was transferred into 10 mL of CAMHB. After vortexing, 50 μL was transferred into each well of a micro-titer plate and incubated for 18 h at 37 °C. The minimum inhibitory concentration (MIC) was determined as the concentration in the first well where visible growth was inhibited. The antibiotics and concentrations tested for each commercial plate are shown in Table 2 and Table 3. The strains ATCC 29213 (*S. aureus*) and ATCC 25922 (*E. coli*) were included as controls. Density and purity controls were included in each sample.

**Table 2:** Minimum inhibitory concentration (mg/L) of 19 *Staphylococcus* spp. isolates from 13 Ugandan pig herds. Values in bold are above the epidemiological cut-off value (ECOFF)

	PEN^**a**^	OXA^**a**^	CEF^**b**^	FOX^**b**^	ENR	FA	ERY	CLI	GEN	NIT	TET	SXT^**1**^
Concentrations in the wells (mg/L)	0.03–1	0.25–1	1–4	0.25–8	0.25–1	0.5–2	0.5–2	0.5–2	1–4	16–64	0.25–4	0.25–4
***S. aureus***												
ECOFF^2^	0.125	2	1	4	nd	0.5	1	0.25	2	32	1	0.25
*S. aureus*^§^	**1**	< 0.25	< 1	4	< 0.25	< 0.5	< 0.5	< 0.5	< 1	< 16	< 0.25	0.25
*S. aureus*	**> 1**	< 0.25	< 1	4	< 0.25	< 0.5	< 0.5	< 0.5	< 1	< 16	< 0.25	**0.5**
*S. aureus*	**> 1**	0.5	< 1	4	< 0.25	< 0.5	**> 2**	< 0.5	< 1	< 16	< 0.25	**0.5**
MRSA	**> 1**	> 1	**2**	**> 8**	< 0.25	< 0.5	**> 2**	< 0.5	< 1	< 16	< 0.25	**0.5**
**Other** ***Staphylococcus*** **spp.**												
ECOFF^3^	0.125^2^	1	1^2^	4^2^	nd	0.5	1	0.25	0.5	32^2^	1	0.25^2^
*S. simulans*	< 0.03	< 0.25	< 1	2	< 0.25	**1**	< 0.5	< 0.5	< 1	< 16	**> 4**	< 0.25
*S. simulans*^#^	< 0.03	< 0.25	< 1	2	< 0.25	0.5	0.5	**0.5**	< 1	< 16	0.5	< 0.25
*S. simulans*	< 0.03	< 0.25	< 1	2	< 0.25	< 0.5	< 0.5	< 0.5	< 1	< 16	**> 4**	< 0.25
*S. simulans*	< 0.03	< 0.25	< 1	4	< 0.25	**1**	< 0.5	< 0.5	< 1	< 16	< 0.25	< 0.25
*S. simulans*	< 0.03	< 0.25	< 1	2	< 0.25	**1**	< 0.5	**1**	< 1	< 16	0.5	**0.5**
*S. chromogenes*^§^	**> 1**	< 0.25	< 1	1	< 0.25	< 0.5	1	< 0.5	< 1	< 16	0.5	**> 4**
*S. chromogenes*	< 0.03	< 0.25	< 1	0.5	< 0.25	< 0.5	**2**	< 0.5	**2**	< 16	0.5	< 0.25
*S. cohnii*^§^	**0.5**	1	< 1	**> 8**	0.5	**> 2**	**> 2**	**1**	< 1	< 16	**> 4**	< 0.25
*S. cohnii*^§^	**0.25**	0.5	< 1	**8**	< 0.25	**> 2**	**> 2**	< 0.5	< 1	< 16	**> 4**	< 0.25
*S. sciurii*^#^	0.06	1	< 1	2	0.5	**> 2**	< 0.5	**1**	< 1	< 16	**> 4**	< 0.25
*S. sciurii*	0.06	1	< 1	2	< 0.25	**2**	< 0.5	< 0.5	< 1	< 16	< 0.25	< 0.25
*S. lentus*^¤^	**0.25**	1	< 1	2	0.5	**2**	< 0.5	**1**	< 1	< 16	0.5	< 0.25
*S. petrasii*	**> 1**	< 0.25	< 1	2	< 0.25	< 0.5	< 0.5	< 0.5	< 1	< 16	0.5	< 0.25
*S. epidermidis*	< 0.03	< 0.25	< 1	2	< 0.25	< 0.5	< 0.5	< 0.5	< 1	< 16	**> 4**	< 0.25
*S. hyicus*	**> 1**	< 0.25	< 1	0.5	< 0.25	< 0.5	< 0.5	< 0.5	< 1	< 16	**> 4**	**1**

Penicillin (PEN), oxacillin + 2% NaCl (OXA), cephalothin (CEF), cefoxitin (FOX), enrofloxacin (ENR), fusidic acid (FA), erythromycin (ERY), clindamycin (CLI), gentamicin (GEN), nitrofurantoin (NIT), tetracycline (TET) and trimethoprim-sulfamethoxazole (SXT)

nd = not defined

MRSA= Methicillin-resistant *S. aureus*

Strains with the same symbol (§, # or ¤) originated from the same herd

^a^Defined as one antibiotic class (penicillins) in the determination of multidrug resistance

^b^ Defined as one antibiotic class (cephalosporins) in the determination of multidrug resistance

^1^ ECOFF for sulfamethoxazole, combined with trimethoprim at a ratio of 1:19

^2^ ECOFF for *S. aureus* is given according to EUCAST [31]

^3^ ECOFF for coagulase-negative staphylococci is given according to EUCAST [31]. If an ECOFF for these were not available, the value has been extrapolated from the ECOFF of *S. aureus*

**Table 3:**
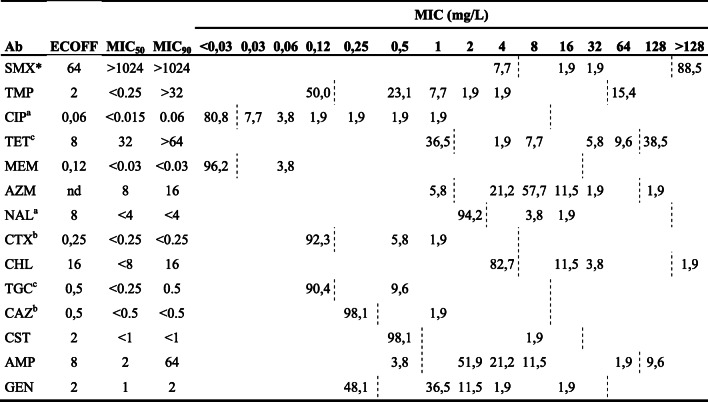
Minimum inhibitory concentrations (MIC; %) of 52 *E. coli* isolates from 19 Ugandan pig herds. Ab indicates the antibiotics tested for in the panels, MIC_50_ and MIC_90_ indicates the concentrations where ≥50 and ≥90% of the isolates, respectively, are inhibited. Values within dashed lines indicate the antibiotic concentrations for which the isolates are tested for each antibiotic

Sulfamethoxazole (SMX), trimethoprim (TMP), ciprofloxacin (CIP), tetracycline (TET), meropenem (MEM), azithromycin (AZM), nalidixic acid (NAL), cefotaxime (CTX), chloramphenicol (CHL), tigecycline (TGC), ceftazidime (CAZ), colistin (CST), ampicillin (AMP) and gentamicin (GEN)

nd = not defined

^a^ Defined as one antibiotic class (quinolones) in the determination of multidrug resistance

^b^ Defined as one antibiotic class (cephalosporins) in the determination of multidrug resistance

c Defined as one antibiotic class (tetracyclines) in the determination of multidrug resistance

The MIC values were compared to epidemiological cut-off values (ECOFF) [31], and isolates with reduced susceptibility were classified as non-wild type. In agreement with the Swedish Veterinary Antibiotic Resistance Monitoring report, non-wild type isolates are referred to as resistant [32]. However, it should be noted that this does not necessarily imply clinical resistance. An isolate was classified as multi-drug resistant (MDR) if it exhibited resistance to at least three classes of antibiotics [33]. The MIC_50_ and MIC_90_ values were calculated for *E. coli* according to Schwarz et al. [33].

## Results

### Antibiotic usage

Seventeen farmers stated that they had treated at least one pig with antibiotics during the last 12 months, mainly using oxytetracycline (OTC) and/or a combination of penicillin and streptomycin, by “injections”. Single farmers also occasionally used trimethoprim-sulfamethoxazole or tylosine injections, sulfamidine tablets or oxytetracycline spray. The number of TO per herd during the previous year varied between one and 13 (median 3), and the number of TO per pig and herd varied between 0.1 and 1.4 (median 0.4). The antibiotics were administered up to five times per TO, but at 31 out of the 65 TO where information was provided, only one treatment was given. In 12 herds, the treatments had been performed by paraveterinarians, i.e. paraprofessional animal health workers, and in five herds by the farmers themselves.

In nine herds, antibiotics had been used as “routine” or “preventive” treatments at least once, while in the other 8 herds, antibiotics had been used solely for the treatment of sick pigs. The three farmers who stated the reasons for these “routine” treatments, reported that it was due to fear of “outbreaks”.

### Bacteriological cultivation and antibiotic resistance

A total number of 50 samples from 83 individual pigs were analyzed for the detection of *Staphylococcus* spp., and 53 individual samples were analyzed for *E. coli*.

#### Staphylococcus spp.

Nineteen strains of *Staphylococcus* spp. were isolated from 18 samples (18/50; 36%), originating from 13 herds. The isolates were identified as *S. aureus* (*n*=4), *S. simulans* (5), *S. cohnii* (2), *S. chromogenes* (2), *S. sciurii* (2), *S. lentus* (1), *S. petrasii* (1), *S. epidermidis* (1) and *S. hyicus* (1). Resistance to penicillin was most commonly found, followed by resistance to fusidic acid, and to tetracycline (Table 2). Resistance to at least one antibiotic substance was found in all 13 herds and eight isolates (42%) from seven (54%) herds exhibited MDR. None of the isolates exhibited resistance to enrofloxacin or nitrofurantoin. One isolate (*S. simulans*) was susceptible to all tested antibiotics. No associations could be made between antibiotic resistance and herd size, treatment regimens or preventive medication strategies (see Additional file 2).

One of the *S. aureus* isolates was confirmed as MRSA by PCR (1/50; 2% of the samples), positive for the *nuc*, *pvl* and *mecA* genes, resulting in an occurrence at herd level of 5% (1/19). The isolate originated from a small farm situated in a village densely populated with both pigs and humans. In this farm, the pigs had received antibiotic treatments from a paraveterinarian on three occasions in the previous year, both as a routine and to treat sick pigs, resulting in 0.7 TO per pig. At two of the treatment occasions, OTC was used, and at one TO, tylosine was used, at all occasions as single injections.

#### E. coli

*Escherichia coli* was isolated from 52 out of 53 samples (98.1%), and the full MIC results of all isolates are given in Additional file 3. Resistance to sulfamethoxazole was most commonly found, followed by resistance to tetracycline and to trimethoprim (Table 3). Four isolates each (7.7%) exhibited resistance to ciprofloxacin and cefotaxime, respectively, while no resistance to carbapenems was found. Resistance to at least one antibiotic substance was found in isolates from at least one pig in all 19 herds. Fourteen isolates (26.9%) from nine herds (47%) exhibited MDR, and five isolates (9.6%) were susceptible to all tested antibiotics. No associations could be made between resistance and herd size, treatment regimens or preventive medication strategies (see Additional file 2).

## Discussion

Antibiotic resistance was commonly found in isolates of both *Staphylococcus* spp. and *E. coli*. It is worrying that nine herds had used antibiotics for the treatment of healthy pigs to e.g. prevent outbreaks, which in Ugandan smallholder pig farms commonly refers to African swine fever, a devastating endemic viral disease that is not susceptible to antibiotics. Further, to avoid the development of resistance, antibiotic treatment of healthy animals should be avoided.

To the best of our knowledge, this is the first report in international literature on the occurrence of MRSA in pigs in Uganda. In line with other studies from other sub-Saharan countries [34, 35], the occurrence seems to be low, since only one MRSA isolate found, carrying both the *mecA* and the virulence factor *pvl* genes. The farm of origin was small (*n*=1.4 sows/year) and had conducted slightly more TO per pig (*n*=0.7) than the average herd in the study. It was also the only herd that had used tylosine in the treatments, and the isolate was also resistant to macrolids. In Uganda, animals and humans sometimes live closely together, and free-ranging pigs are common [19]. Transfer of bacteria and/or resistance genes between humans and pigs is thus not unlikely. However, since no further genetic characterization of the isolate was performed, the molecular epidemiology and origin of the isolate is unknown.

The high number of antibiotic-resistant isolates of both *Staphylococcus* spp. and *E. coli*, in particular displaying resistance to tetracycline, penicillins and sulfonamides, are important findings since these antibiotics are commonly used to treat livestock in Uganda [36] and are critically or highly important to both human and veterinary medicine [37, 38]. Further, resistance to other high priority critically important antimicrobials such as macrolides, quinolones and third generation cephalosporins was found in several isolates. While no further analyzes were performed, the results also suggest the possible presence of extended spectrum β-lactamase (ESBL) producing *E. coli*.

No associations between antibiotic resistance and herd size or treatment regimens were detected for any of the bacteria, regardless of whether the animals had been treated routinely for prophylactic purposes, or upon clinical signs of disease only, however, the sample size was small. Thus, future studies may include more herds to increase the possibility of detecting any such associations, and, if possible, also include investigations on bacterial isolates collected from the farmers and animal care-takers. Nevertheless, the number of resistant *E. coli* isolates in this study were slightly lower than previously reported in studies from other low- and middle-income countries [39]. However, different sampling and culturing methods and differences in the presentation of the results make the outcomes from various studies difficult to compare [21, 33]. The number of isolates from each species of *Staphylococcus* was too low to allow any conclusions to be drawn on species-level, however, *S. cohnii* seems to be more prone to develop resistance as compared to e.g. *S. simulans*, as previously noted by Ouba et al. [40].

In some of the herds where resistance to tetracycline in *E. coli* was detected, tetracycline resistance has previously been demonstrated in isolates from clinical cases of post-weaning diarrhea [22]. Further, isolates of *S. sciuri* from a healthy pig in this study exhibited similar antibiotic resistance pattern as an isolates of *S. sciuri* previously recovered from a clinical case of exudative epidermitis in the same herd (data not shown).

In the present study, minor practical-methodological challenges were faced such as slightly fluctuating incubator temperatures, pipettes with unclear calibration status and autoclaved pipette tips. Thus, it cannot be excluded that this might have affected the results, however, the antibiotic-susceptibility profiles of the control strains were within the pre-determined limits. Further, in the interpretation of the levels of sulfamethoxazole resistance in *E. coli,* growth was only assessed as “growth” or “no growth”, whereas the method allows for up to 20% growth to be assessed as negative according to the manufacturer’s instructions. The level of resistance to trimethoprim was much lower than to sulfamethoxazole, as previously noted in a study of other *Enterobacteriacae* in Uganda [41]. This could be related to either the potential methodological short-comings in the analysis of sulfamethoxazole as described above, or resulting from co-selection as sulphonamide resistance may be linked to other types of resistance, e.g. through association with class 1 integrons [42]. Further, it might possibly also be explained by the common use of sulfonamides without being combined with trimethoprim [36]. The use of sulfonamide tablets was only mentioned by one farmer, but it is possible that others also used them but failed to report this usage during the interviews, as these were not given as “injections” and may thus not have been regarded as a treatment. In support of this hypothesis, many farmers also reported the use of various drugs, mainly anthelmintics and endectocides, when asked about their use of antibiotics. This lack of knowledge about drugs might have confounded the results, if such treatment occasions were misinterpreted as e.g. preventive antibiotic use. However, since the interviews were complemented by longitudinal data and the names of the drugs were retrieved in most cases, these results were likely reliable.

## Conclusion

In conclusion, antibiotic resistance to frequently used and critically or highly important antibiotics to both human and veterinary medicine, such as e.g. tetracycline, was commonly found in isolates of *Staphylococcus* spp. and *E. coli* from Ugandan pigs. Further, several farmers used antibiotics for the purpose of “preventive” or “routine” treatments, however, this did not seem to influence the level of resistance in single herds. Methicillin-resistant *S. aureus* carrying the *mecA* and the virulence factor *pvl* genes were found in one of the investigated pigs.

## Supplementary Information

**Additional file 1.** Structured interview. Structured interview conducted in September 2019 in all studied herds, on the antibiotic usage during the last 12 months.

**Additional file 2.** Statistics. Herd level statistical analyzes using one-tailed Fisher’s exact test, comparing resistance (Yes/No) to any of the antibiotics included in the panels, resistance to single selected antibiotic substances (tetracycline and penicillin/ampicillin) and the presence of multi-drug resistance, to the herd size and treatment regimens.

**Additional file 3. **Antibiotic resistance of *E. coli.* Minimum inhibitory concentrations (mg/L) of the 52 *E. coli* isolates.

## Data Availability

The datasets generated and/or analyzed during the current study are available from the corresponding author on reasonable request.
