# Supplementary material for: The presence of antibiotic-resistant Staphylococcus spp. and Escherichia coli in smallholder pig farms in Uganda
Source: BMC Vet Res. 2021 Jan 18;17:31. doi: 10.1186/s12917-020-02727-3 (PMC7814613; doi:10.1186/s12917-020-02727-3)
Supplement: Supplementary file 1 — Additional file 1. Structured interview. Structured interview conducted in September 2019 in all studied herds, on the antibiotic usage during the last 12 months. [file 12917_2020_2727_MOESM1_ESM.docx]

# Structured interview

Farm number:

Date of visit:

1. Have the pigs received any medical treatment with antibiotics during the last year?

Yes No

*If yes, continue with question 2. Otherwise end of questionnaire.*

1. How often or how many times have the pigs received treatment?
2. Why did you treat the pigs with antibiotics?

As a routine treatment To treat sick pigs Both

1. *If sick pigs*; What symptoms did you treat?
2. *If routine treatment*;
   1. Why do you treat the pigs?
   2. How often do the pigs receive routine treatments?
3. Did you or a veterinarian/paraveterinarian treat the pigs?

Veterinarian/paraveterinarian Farmer him/herself

1. *If the farmer him/herself conducted the treatment*;
   1. On what grounds do you base the treatments?
   2. How did you choose the type of antibiotic to use?
2. What dosage of antibiotics have the pigs received?
3. For how long were each pig treated?

*e.g. once per day for three days*

1. Where/how do you buy or get hold of antibiotics?
2. What kind of antibiotics have you used (*ie drug names*)?
3. Other comments on antibiotic treatment;
