# Supplementary material for: The presence of antibiotic-resistant Staphylococcus spp. and Escherichia coli in smallholder pig farms in Uganda
Source: BMC Vet Res. 2021 Jan 18;17:31. doi: 10.1186/s12917-020-02727-3 (PMC7814613; doi:10.1186/s12917-020-02727-3)
Supplement: Supplementary file 2 — Additional file 2. Statistics. Herd level statistical analyzes using one-tailed Fisher’s exact test, comparing resistance (Yes/No) to any of the antibiotics included in the panels, resistance to single selected antibiotic substances (tetracycline and penicillin/ampicillin) and the presence of multi-drug resistance, to the herd size and treatment regimens. [file 12917_2020_2727_MOESM2_ESM.docx]

# Statistics

| Statistics performed using Fisher's exact test, one-tailed (<https://www.graphpad.com/quickcalcs/contingency2/>) | | | | | |  |
| --- | --- | --- | --- | --- | --- | --- |
|  | ***E. coli***  **(n=19)** | | | ***Staphylococcus* spp. (n=13)** | | |
| **Resistance to at least one type of antibiotic** | | | | | | |
|  | Yes | No | p-value | Yes | No | p-value |
| **Herd size** |  |  | 1,00 |  |  | 1,00 |
| Small | 14 | 0 |  | 14 | 0 |  |
| Large | 5 | 0 |  | 5 | 0 |  |
| **Antibiotic treatment** |  |  | 1,00 |  |  | 1,00 |
| Yes | 17 | 0 |  | 17 | 0 |  |
| No | 2 | 0 |  | 2 | 0 |  |
| **Treatment occasions (median 3)** |  |  | 1,00 |  |  | 1,00 |
| >3 | 5 | 0 |  | 5 | 0 |  |
| ≤3 | 11 | 0 |  | 11 | 0 |  |
| **Treatment occasions per pig (median 0.4)** | |  | 1,00 |  |  | 1,00 |
| >0.4 | 8 | 0 |  | 8 | 0 |  |
| ≤0.4 | 8 | 0 |  | 8 | 0 |  |
| **Treatment reason** |  |  | 1,00 |  |  | 1,00 |
| Only sick pigs | 8 | 0 |  | 8 | 0 |  |
| Routine treatments and sick pigs | 9 | 0 |  | 9 | 0 |  |
| **Resistance to at least three types of antibiotics (multi-drug resistance)** | | | | | | |
|  | Yes | No |  | Yes | No |  |
| **Herd size** |  |  | 0,14 |  |  | 0,07 |
| Small | 5 | 9 |  | 3 | 6 |  |
| Large | 4 | 1 |  | 4 | 0 |  |
| **Antibiotic treatment** |  |  | 1,00 |  |  | 0,19 |
| Yes | 8 | 9 |  | 7 | 4 |  |
| No | 1 | 1 |  | 0 | 2 |  |
| **Treatment occasions** |  |  | 0,63 |  |  | 1,00 |
| >3 | 3 | 2 |  | 3 | 2 |  |
| ≤3 | 6 | 8 |  | 4 | 4 |  |
| **Treatment occasions per pig** |  |  | 0,07 |  |  | 0,13 |
| >0.4 | 2 | 7 |  | 2 | 6 |  |
| ≤0.4 | 7 | 3 |  | 5 | 2 |  |
| **Treatment reason** |  |  | 0,35 |  |  | 1,00 |
| Only sick pigs | 5 | 3 |  | 3 | 2 |  |
| Also routine treatments | 3 | 6 |  | 4 | 1 |  |
| **Resistance to tetracycline** | | | | | | |
|  | Yes | No |  | Yes | No |  |
| **Herd size** |  |  | 0,13 |  |  | 1,00 |
| Small | 8 | 6 |  | 4 | 5 |  |
| Large | 5 | 0 |  | 2 | 2 |  |
| **Antibiotic treatment** |  |  | 1,00 |  |  | 1,00 |
| Yes | 12 | 5 |  | 6 | 5 |  |
| No | 1 | 1 |  | 1 | 1 |  |
| **Treatment occasions** |  |  | 0,13 |  |  | 0,59 |
| >3 | 5 | 0 |  | 3 | 2 |  |
| ≤3 | 8 | 6 |  | 3 | 5 |  |
| **Treatment occasions per pig** |  |  | 0,35 |  |  | 1,00 |
| >0.4 | 5 | 4 |  | 3 | 5 |  |
| ≤0.4 | 8 | 2 |  | 3 | 4 |  |
| **Treatment reason** |  |  | 0,29 |  |  | 1,00 |
| Only sick pigs | 7 | 1 |  | 3 | 2 |  |
| Also routine treatments | 5 | 4 |  | 2 | 3 |  |
| **Treatment with oxytetracycline** |  |  | 1,00 |  |  | 0,45 |
| Yes | 9 | 4 |  | 4 | 6 |  |
| No | 4 | 2 |  | 2 | 0 |  |
| **Resistance to ampicillin (*E. coli*) or penicillin (*Staphylococcus* spp.)** | | | | | | |
|  | Yes | No |  | Yes | No |  |
| **Herd size** |  |  | 1,00 |  |  | 0,56 |
| Small | 3 | 11 |  | 4 | 6 |  |
| Large | 1 | 4 |  | 3 | 1 |  |
| **Antibiotic treatment** |  |  | 0,39 |  |  | 1,00 |
| Yes | 3 | 14 |  | 6 | 5 |  |
| No | 1 | 1 |  | 1 | 1 |  |
| **Treatment occasions** |  |  | 0,27 |  |  | 0,59 |
| >3 | 2 | 3 |  | 2 | 3 |  |
| ≤3 | 2 | 12 |  | 5 | 3 |  |
| **Treatment occasions per pig** |  |  | 0,58 |  |  | 1,00 |
| >0.4 | 1 | 8 |  | 4 | 4 |  |
| ≤0.4 | 3 | 7 |  | 4 | 3 |  |
| **Treatment reason** |  |  | 0,08 |  |  | 0,52 |
| Only sick pigs | 3 | 5 |  | 4 | 1 |  |
| Also routine treatments | 0 | 9 |  | 2 | 3 |  |
| **Treatment with penicillin**  **in combination with streptomycin** | | | 0,56 |  |  | 0,52 |
| Yes | 2 | 4 |  | 3 | 1 |  |
| No | 2 | 11 |  | 2 | 3 |  |
